# Association between male pattern baldness and testicular germ cell tumor: a meta-analysis

**DOI:** 10.1186/s12885-018-5197-5

**Published:** 2019-01-11

**Authors:** Jiatong Zhou, Shuai Xia, Tao Li, Ranlu Liu

**Affiliations:** The Second Hospital of Tianjin Medical University, Department of Urology, Tianjin Institute of Urology, No. 23 Pingjiang Road, Hexi District, Tianjin, People’s Republic of China

**Keywords:** Male pattern baldness, Meta-analysis, Testicular germ cell tumor, Risk

## Abstract

**Background:**

The relationship between male pattern baldness and incidence of testicular cancer remains inconclusive. Hence, we performed the present meta-analysis based on all eligible case–control studies.

**Methods:**

A comprehensive literature search was performed in July 30th 2018 based on PUBMED, EMBASE and Web of science database. Pooled odds ratio(OR) and its 95% confidence intervals (95% CIs) was calculated with a DerSimonian and Laird random-effects.

**Results:**

The pooled results were included in this meta-analysis. Overall, We have demonstrated statistically signification between baldness(any pattern) and testicular cancer was identified (OR: 0.61, 95% CI:0.50–0.74). There was no obvious heterogeneity across included studies (*P* = 0.22 for heterogeneity, I^2^ = 30%). When subgroup analysis by types of baldness, We found a statistically significant association was observed that baldness(I-VII) might become a protective factor for the risk of testicular germ cell tumor(TGCT). There was no definite connection between alopecia and the different types of TGCT.

**Conclusion:**

Individuals with any pattern baldness may have a decreased risk of testicular cancer, all of analyses studies are warranted to confirm our preliminary findings. According to subgroup analysis of different hair loss grades, we found that 2 stage(II) hair loss can decrease more strongly testicular cancer risk than any other grades. Despite of our findings, We still need further researches to advance knowledge in this field.

## Background

Male pattern baldness, also known as Androgenetic alopecia (AGA), is an inherited pattern of baldness hormone-regulated by the testosterone metabolite dihydrotestosterone characterized by follicular androgen sensitivity and follicular miniaturization [1]. While inhibition of Dihydrotestosterone (DHT) production slows the progression of male pattern baldness (MPB) [2]. The onset of androgenetic alopecia is extremely variable, and appears to be determined by the presence of sufficient circulating androgens and the degree of genetic predisposition. And also androgenetic alopecia is a polygenic mode of Inheritance [1]. Moreover, its frequency increases with age and affects up to 80% Caucasian men and 42% of women [3]. AGA is a common disorder affecting almost 50% of men throughout their lifetime [4] androgen status does not decline with aging among older men who remain in excellent (asymptomatic) health [5]. So, We have the reason to believe that AGA plays an important role in the pathomechanism of some androgen related tumors in men. AGA shares a substantial biological basis with numerous other human phenotypes [6]. This may have major implications in terms of the evaluation of MPB as an early prognostic marker for different phenotypes such as prostate cancer(PC), sudden cardiacarrest or neurodegenerative disorders. Testicular cancer is a relatively rare tumor representing < 2% of all malignancies in men. Nevertheless, it is the most common tumor among young males in Europe and North America [7] the only well-documented risk factors are cryptorchidism, prior history of TGCT, and family history of TGCT [7]. The fact that testicular cancer is hormone-dependent is supported by its rapid increase in incidence starting at pubertal age. In addition, late age at puberty has been inversely associated with the risk of testicular cancer [3]. In androgenetic alopecia, hormonal signals lead to change in genes regulating hair follicle size and cycling and there is a gradual diminution of the hair follicle [8]. AGA and testicular cancer share some biological and epidemiologic risk factors: aging, genetics inheritance, and androgenetic influence. Although Liang et al. [9] conducted a meta-analysis to reveal the relationship between AGA and testicular cancer in 2018,we analyzed the data about the risk of testicular cancer from a new case-control study reported by Lee et al. [10] that men with AGA may not be associated with testicular cancer. This current analysis we obtained is different from the previous statistic result reported by Liang et al. [9].

As a result, we conducted a systematic review and a comprehensive meta-analysis in order to further investigate the issue and identify potential sources of heterogeneity that might be confounders that have affected some existing conclusions.

## Methods

### Literature search

**Fig. 1:**
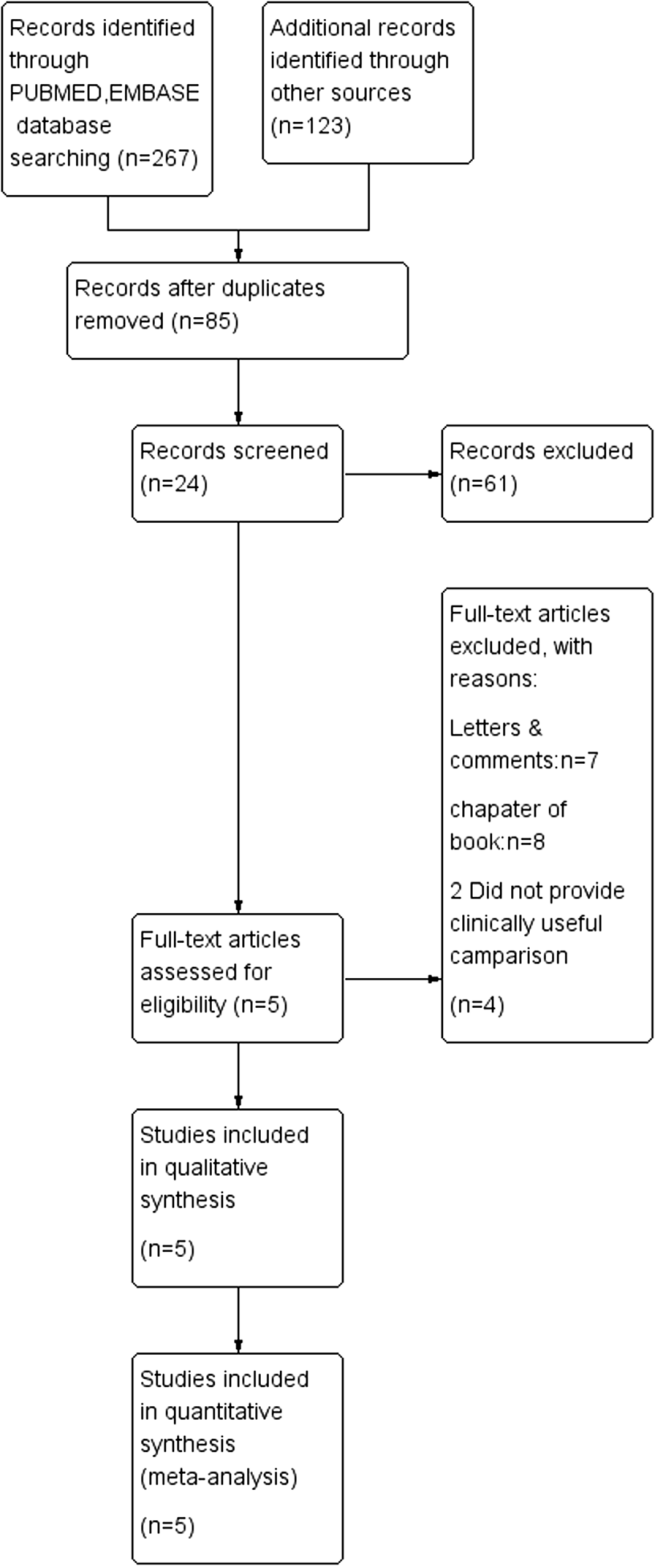
Flow diagram of included studies for this meta-analysis

A comprehensive literature search was performed in JULY 30th 2018 based on PubMed, EMBASE and Web of Science databases with the following search algorithm: (“baldness” or “alopecia” or “hair loss”) and (“testicular cancer” or “testicular neoplasm” or“testicular germinal cell tumor”). In addition, the reference lists of retrieved articles and related reviews were checked to identify any potential relevant studies. No language or publication date restrictions were adopted. The flow diagram was presented in Fig. 1. The present systematic review and meta-analysis was designed, performed, and reported in accordance with the standards of quality for reporting meta-analysis.

### Selection criteria

Articles included in this study had to meet all the following criteria: the exposure of interest was male pattern baldness; the outcome of interest was risk of testicular cancer; study design was nested case–control, or case-control; and the risk estimates with their corresponding 95% CIs were reported or sufficient datas were provided to calculate them.

### Quality assessment

Newcastle–Ottawa Scale (NOS) was introduced to evaluate the quality of included studied by two independent reviewers(Tao L, Shuai X). NOS is a 9-star system, which includes 3 dimensions: selection (4 items), comparability (1 item), and exposure/outcome (3 items). Each item represents 1 point, except for comparability (2 points). A study with ≥7 points was considered as a high-quality study.

### Data extraction

Data were extracted independently by the same 2 reviewers (Tao L, Shuai X) and any discrepancies were resolved by discussion. The following data from each included study were recorded: first author’s surname, the country where the study was conducted, study design, publication year, sample size and number of cases, types of baldness, fully adjusted risk estimates with their corresponding 95% confidence intervals (95% CIs), and matched or adjusted variables in the study design or statistical analysis.

### Statistical methods

For studies that reported separate effect size estimates for different types of baldness, we combined these risk estimates within each study, weighted by inverse of the variance [7]. Subgroup analyses were performed based on types of baldness, types of testicular cancer. Heterogeneity across included studies was assessed with the CochranQtest (the level of significance was set at 0.1) [11]. The I^2^ score was also used to determine the degree of heterogeneity(I^2^ < 50%, no obvious heterogeneity; I^2^ > 50%, large or extreme heterogeneity) [11]. Galbraith plot [12] was introduced to identify the studies that contributed to the heterogeneity and meta-analysis was performed again after removing these studies.

In sensitivity analysis, meta-analysis was reconducted after omitting each study in turn. Potential publication bias was evaluated with Begg’s test and Egger’s test [13]. Statistical analyses were completed with Reviewer Manager 5.3. All analyses of this meta-analysis were based on previous published studies, and this meta-analysis did not have original data. Thus, no ethical approval and patient consent are required.

## Results

### Study searches and characteristics

The detailed process of literature search is shown in Fig. 1. A total of 5 eligible studies [10, 14–17] were finally included in the present meta-analysis. These studies were carried out in the following geographical regions: USA (*n* = 2), Italy (*n* = 1), Greece (*n* = 1), Nation (*n* = 1), There were 5 case–control studies [10, 14–17], which were published between 1997 and 2018. Information on exposure (male pattern baldness) was collected by face-to-face interview or self-reported questionnaire. Study outcome (testicular tumor) was histopathologically proved in most of the included studies. Quality scores evaluated by the Newcastle–Ottawa Scale (NOS) ranged from 5 to 7. The main characteristics of all included studies have been summarized in Table 1.

**Table 1:** Characteristics of included studies: androgenic alopecia and TGCT risk

Study	Country	Year	Type of study	Sample		Association with TGCT, OR[95%]
				Cases	Controls	Any baldness
Lee	Korea	2018	case-control	188	668,614	0.87[0.59,1.31]
Moirano	USA	2016	case-control	253	455	0.64[0.45,0.91]
Trabert	USA	2011	case-control	187	148	0.50[0.32,0.77]
Farzana	Italy	2002	case-control	159	136	0.51[0.32,0.82]
Petridou	Greece	1997	case-control	97	198	0.45[0.26,0.77]

In this table, Abbreviation: *TGCT* Testicular germ cell tumor, *OR* Odd rate, All the literature studies are case-control studies, and each study has corresponding OR values and confidence intervals. Lee’s study has the largest population and the largest OR values

### Association between any AGA and testicular cancer

**Fig. 2:**
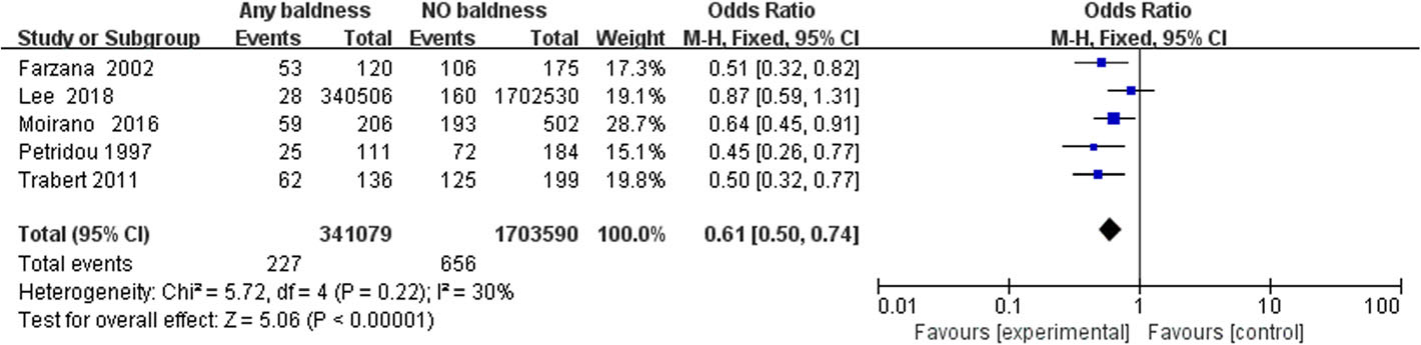
Forest plots of any AGA and the risk of TGCT incidence for case-control studies. Abbreviation: *TGCT* Testicular germ cell tumor, *AGA* Androgenetic alopecia, According to the incidence of testicular cancer in alopecia and non-alopecia population, the total OR value was 0.61, *P* < 0.001, and the I^2^ was 30% < 50%. The overall study has statistical significance

**Fig. 3:**
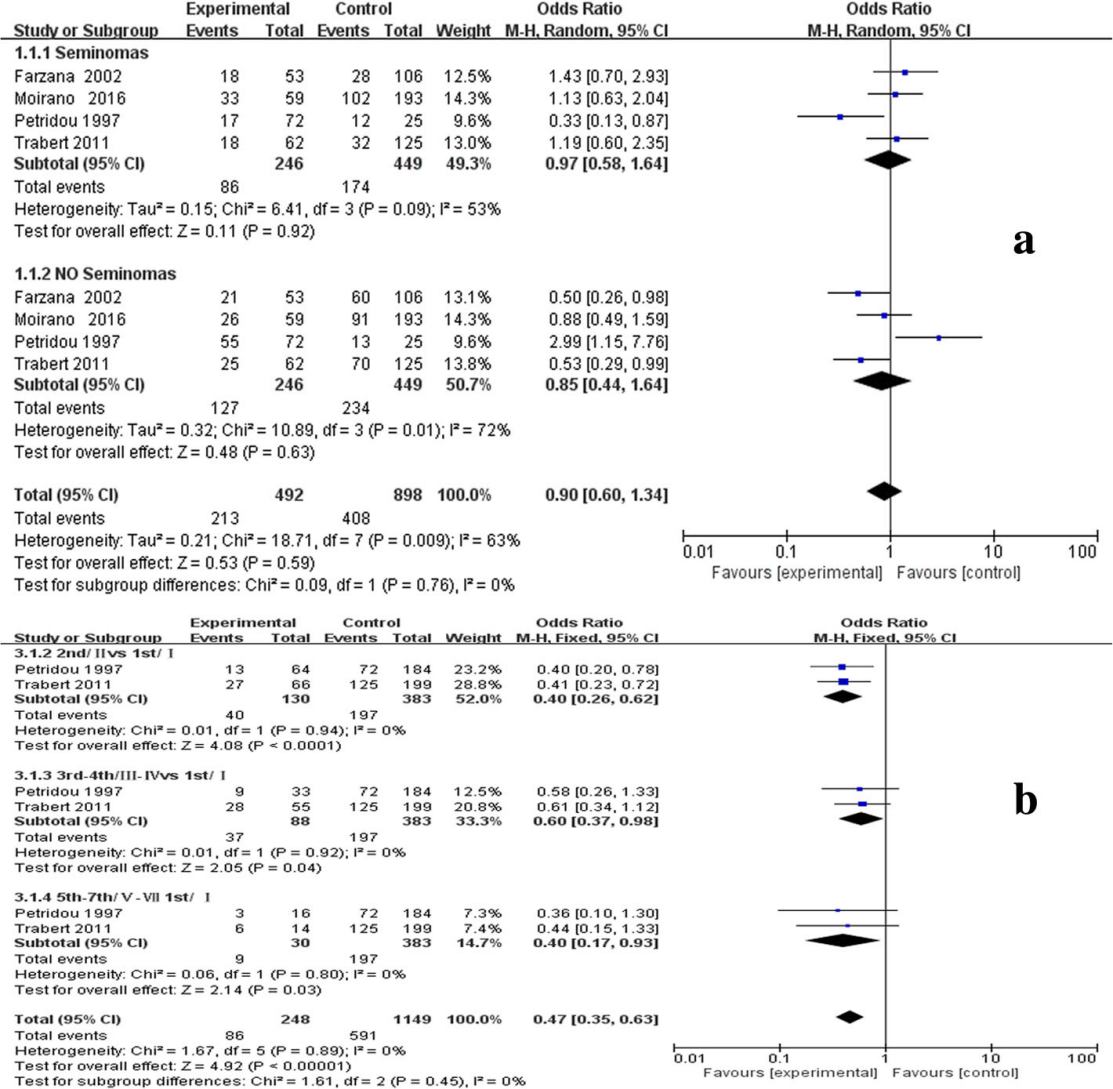
Differential subgroup analysis of TGCT and AGA: **a** for different types of TGCT with any AGA; **b** for any TGCT with different types of AGA. For any type of alopecia (**a**), we found no significant difference in the risk of different types of alopecia and testicular cancer(OR = 0.90;95% CI:0.60–1.34; *P* = 0.67). For any type of TGCT, Grade II alopecia has statistical significance for the risk of testicular cancer(OR = 0.4; 95% CI:0.26–0.62; *P* < 0.001)

We examined the relationship between any pattern baldness and the risk of TGCT using data from 5 studies [10, 14–17]. There was significant result that men with any pattern baldness could have negative association with testicular cancer (OR = 1.05;95% CI:0.50–0.74; *P* < 0.00001) (Fig. 2). When the studies were stratified by histological subtypes, the results were consistent and we found baldness has no significant connection with the risk of different types of TGCT (OR = 0.90;95% CI:0.60–1.34; *P* = 0.67) (Fig. 3a). When the studies were stratified by different degrees of baldness on the basis of the Hamilton-Norwood scale, pattern baldness at 2nd stage(II) was more negatively correlated with TGCT risk compared to that at other stages (OR = 0.4; 95% CI:0.26–0.62; *P* < 0.001) (Fig. 3b).

### Sensitivity analysis and publication bias

**Fig. 4:**
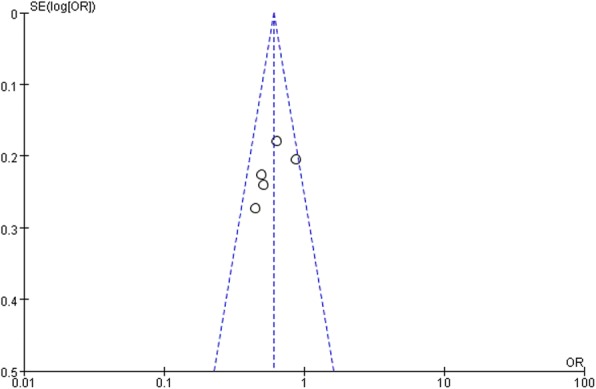
Begg’s funnel plots for publication bias of the relative risk of TGCT incidence for all case-control reports. There was no obvious publication bias in the our literatures

Sensitivity analysis confirmed that no individual study influenced the overall results. There was no evidence of publication bias in this meta-analysis indicated by the Begg’s funnel plot and Egger’s tests [13] (Fig. 4).

## Discussion

This systematic review and meta-analysis involved a total of 884 cases from 5 observational studies [10, 14–17]. Our study included the largest sample as far as we know. From the comprehensive data we analyzed, we concluded that men with any pattern baldness may decreased the risk of testicular cancer, which means that AGA could be a protective factor in the cancer of testicle. In the subgroup between the type of baldness, we discovered man with AGA at grade 2(II) may have less risk than other stages, In addition, we found that there was no significant relationship between types of testicular tumor and AGA. Before we did our study, a systematic analysis of the relationship between alopecia and testicular cancer had been discussed. That mata-analysis included 4 case-control reports from 1997 to 2016 which reported that man with baldness could have less risk of testicular cancer compared with no baldness. And they found that baldness is significant inversly related with nonseminoma. However, from the recently report by Lee et al., we could not see any obvious connection between baldness and the risk of TGCT. The mechanism of alopecia in the risk of testicular cancer is still unclear. While in another discussion about the association between AGA and prostate diseases, especially prostate cancer which has been studied thoroughly, in Zhou et al’s study [18], which included 1138 incident prostate cancer cases, they concluded that men with frontal plus moderate vertex baldness at age 45 years has an increased risk of aggressive prostate cancer. This result provided evidence that we have reason to believe that hair loss is associated with male reproductive system tumors. In Petridou et al. [14] research,they had studied different factors on the risk of testicular cancer including age,body mass index, baldness, height and so on. Among these factors,they discovered a conclusion that Baldness is inversely associated with testicular cancer (*p* = 0.04). This was the first time that baldness had been linked to testicular cancer in a case-control study. However,in this study report,it had some limitations, including that it only considered the relationship between different types of alopecia and testicular cancer,but not included the subtypes of testicular cancer were not presented. In Farzana et al. [15], they found that no significant association between baldness and this cancer, and also found that seminoma, which has a known older age at diagnosis than nonseminoma. In Trabert et al. ‘s report [16], they got same result comparied with Petridou [14] and Farzana [15], in addition, their findings of inversly association between baldness and testicular cancer, especially among men with nonseminoma, which conflicted with Moirano et al’s report [17] that this association between AGA and TGCT was more stronger among seminoma. so we still need a large number correlation studies to clarify the relationship between hair loss and testicular cancer. Besides the male pattern baldness, there might be some related factors which are significantly related with testicular cancer,like race, age, Body Mass Index (BMI), young adult African Americans who have higher levels of circulating testosterone than their white counterparts [19], according to Depue et al. [20] they got the insight that severe acne at puberty, which is thought to reflect increased levels of androgens, was inversely associated with the risk of testicular cancer. So, we can consider African Americans may have a lower risk of testicular tumor. As far as we concerned, as proxies for high androgen levels or high androgen sensitivity, alopecia and acne require a deeper understanding of the mechanisms of androgen action and its impact on testicular cancer. AGA could be used as a signal for sex hormone metabolism, hormone secretion and human androgen sensitivity. Men with AGA have higher levels of dihydrotestosterone within the hair follicles and its blockage helps to slow the progression of baldness [21]. It has been hypothesized that puberty maybe a period of development during which endogenous hormonal factors increase the risk of TGCT [22], It has been reported that circulating testosterone, DHT, and E2 declined gradually during male aging [8]. androgen activity through multi-step signaling process in pathophysiology of AGA [23]. In addition, androgen receptor and androgen metabolic pathway genetic variation studies showed that Ser312-Asn polymorphism of the luteinizing hormone receptor was linked to an decreased relative risk of TGCT [24]. These findings provide sufficient evidence for the effect of AGA on testicular cancer.

In the similar study reported by Liang et al. [9], we got the same result that AGA exposure is inversely related to the risk of TGCT and men with 2nd stage baldness could have lower incidence of testicular. However,about the histological subgroup, we got no closed relationship between AGA and type of testicular cancer, this is in conflict with the view authors had mentioned that the inverse relationship was more predominant among nonseminoma with less heterogeneity. And also,in another report written by Lee et al. [10], we did not find a link among hair loss and testicular cancer, although they analyzed a large number of cases.From these related studies, we have suggested that hormonal related factors was significantly inversely related to the risks of TGCT. However, because of the low incidence of testicular tumors in the population, we do not have sufficent case to analyze, which may limit our results. In addition, other factors like race, age, hight also affect the heterogeneity.

## Conclusions

As an important organ that maintains male charecteristics, the human testes are prime targets for hormonal modulation. We found evidence of association between AGA at any age and a significant decreased risk of testicular cancer based on meta-analysis of case-control studies,in histological subgroup analysis, we explored that the second level of hair loss is more benificial for reducing the risk of TGCT. This conclusion may suggest that AGA or men with baldness may be the protective factor for the TGCT. AS far as we concerned, our study includes only 5 case-control reports and there are some divergent views in these documents. Further andomized controlled trials studies should be required to confirm the potential relationship between AGA and TGCT.

## Abbreviations

AGA: Androgenic alopecia
CIs: Confidence intervals
DHT: Dihydrotestosterone
HRs: Hazard ratios
MPB: Male pattern baldness
NOS: Newcastle-Ottawa Scale
ORs: Odds ratios
PC: Prostate cancer
TGCT: Testicular germ cell tumor
